# Special Issue: Materials for Electrochemical Capacitors and Batteries

**DOI:** 10.3390/ma10040438

**Published:** 2017-04-22

**Authors:** Jian-Gan Wang, Bingqing Wei

**Affiliations:** 1 State Key Laboratory of Solidification Processing, Center for Nano Energy Materials, School of Materials Science and Engineering, Northwestern Polytechnical University and Shaanxi Joint Lab of Graphene (NPU), Xi’an 710072, China; 2 Department of Mechanical Engineering, University of Delaware, Newark, DE19716, USA

**Keywords:** Li-ion batteries, electrode materials, asymmetric supercapacitor, Li-ion capacitor, Li-sulfur batteries

## Abstract

Electrochemical capacitors and rechargeable batteries have received worldwide attention due to their excellent energy storage capability for a variety of applications. The rapid development of these technologies is propelled by the advanced electrode materials and new energy storage systems. It is believed that research efforts can improve the device performance to meet the ever-increasing requirements of high energy density, high power density and long cycle life. This Special Issue aims to provide readers with a glimpse of different kinds of electrode materials for electrochemical capacitors and batteries.

## 1. Introduction

The rapid fossil depletion and the serious environmental problems have sparked unprecedented research efforts in developing novel energy storage technologies from sustainable and renewable energy resources in recent years [1,2,3]. Among the different technologies, electrochemical capacitors (also called supercapacitors) and rechargeable batteries are the most attractive energy storage systems that may find widespread applications ranging from consumer electronics, electric vehicles to large-scale smart utility grids [4,5,6]. However, the-state-of-the art power sources cannot meet the ever-increasing demands for high energy density, high power density and long cycle life [7]. Electrode materials play a significant role in determining the energy storage performance [8,9]. In addition, exploring energy storage devices based on new chemistry/configuration is also an effective approach to enhance the performance [10,11]. The booming development in these fields motivated us to organize this Special Issue, which aims to address current and future advancements in all aspects of materials science and engineering and their applications for supercapacitors and rechargeable batteries.

This Special Issue covers seven research articles, including four papers on Li-ion batteries, one paper on Li-sulfur batteries, one paper on Li-ion capacitors and one paper on asymmetric supercapacitors. With regard to the anode materials for Li-ion batteries, Wang et al. developed a low-cost yet high-performance anode material of MoS_2_/bio-mass-derived carbon composite that can deliver a specific capacity of 820 mAh g^−1^ [12], while Jeong et al. reported a binder-free anode of mesoporous carbon nanotube–carbon nanofiber prepared by electrospinning [13]. In the field of cathode materials for Li-ion batteries, Wang et al. explored a three-dimensional V_2_O_5_ hollow structure through a novel solvothermal synthesis strategy, and comparesd the Li-ion storage performance of V_2_O_5_ annealed at different temperatures [14]. Wen et al. reported a novel deep eutectic solvent method to synthesizing LiMnPO_4_/C nanorods, which exhibited high specific capacity, excellent rate capability and cycling stability [15]. As for the Li-sulfur batteries, Yang et al. introduced two kinds of electrospun carbon nanofiber (CNF) for anode and cathode interlayers to greatly improve the cycling stability even with sulfur loading as high as 80% of the total mass of the cathode [16]. Li-ion capacitors are promising for filling up the performance gap between Li-ion batteries and electrochemical capacitors. Huang et al. developed a high performance Li-ion capacitor with both electrodes prepared from Sri Lanka graphite ore. The device can deliver maximum energy/power densities of 86 Wh kg^−1^/7.4 kW kg^−1^, which holds great promise for practical application due to the low-cost raw materials and industrially feasible production [17]. Asymmetric configuration is a promising way of enlarging the operating voltage of electrochemical capacitors. Huang et al. prepared mesoporous Mn_1.5_Co_1.5_O_4_ spinel films on Ni foam by direct electrodeposition, which was used as a positive electrode to couple an activated carbon to build an asymmetric supercapacitor [18]. An enlarged stable operating voltage of 2.0 V was obtained, enabling a high energy density of 27.6 Wh kg^−1^ while maintaining outstanding cycling performances.

This Special Issue covers seven research articles, including four papers on Li-ion batteries, one paper on Li-sulfur batteries, one paper on Li-ion capacitors and one paper on asymmetric supercapacitors. With regard to the anode materials for Li-ion batteries, Wang et al. developed a low-cost yet high-performance anode material of MoS_2_/bio-mass-derived carbon composite that can deliver a specific capacity of 820 mAh g^−1^ [12], while Jeong et al. reported a binder-free anode of mesoporous carbon nanotube–carbon nanofiber prepared by electrospinning [13]. In the field of cathode materials for Li-ion batteries, Wang et al. explored a three-dimensional V_2_O_5_ hollow structure through a novel solvothermal synthesis strategy, and compared the Li-ion storage performance of V_2_O_5_ annealed at different temperatures [14]. Wen et al. reported a novel deep eutectic solvent method to synthesizing LiMnPO_4_/C nanorods, which exhibited high specific capacity, excellent rate capability and cycling stability [15]. As for the Li-sulfur batteries, Yang et al. introduced two kinds of electrospun carbon nanofiber (CNF) for anode and cathode interlayers to greatly improve the cycling stability even with sulfur loading as high as 80% of the total mass of the cathode [16]. Li-ion capacitors are promising for filling up the performance gap between Li-ion batteries and electrochemical capacitors. Huang et al. developed a high performance Li-ion capacitor with both electrodes prepared from Sri Lanka graphite ore. The device can deliver maximum energy/power densities of 86 Wh kg^−1^/7.4 kW kg^−1^, which holds great promise for practical application due to the low-cost raw materials and industrially feasible production [17]. Asymmetric configuration is a promising way of enlarging the operating voltage of electrochemical capacitors. Huang et al. prepared mesoporous Mn_1.5_Co_1.5_O_4_ spinel films on Ni foam by direct electrodeposition, which was used as a positive electrode to couple an activated carbon to build an asymmetric supercapacitor [18]. An enlarged stable operating voltage of 2.0 V was obtained, enabling a high energy density of 27.6 Wh kg^−1^ while maintaining outstanding cycling performances.
